# Influence of Factors Relating to Sex and Gender on Rank List Decisions and Perceptions of Residency Training: Survey Study

**DOI:** 10.2196/33592

**Published:** 2022-04-05

**Authors:** Ryan Gibney, Christina Cantwell, Alisa Wray, Megan Boysen-Osborn, Warren Wiechmann, Soheil Saadat, Jonathan Smart, Shannon Toohey

**Affiliations:** 1 Department of Emergency Medicine University of California, Irvine Medical Center Orange, CA United States

**Keywords:** residency, sex, gender, graduate medical education, emergency medicine, residents, program leadership, rank list

## Abstract

**Background:**

Females make up more than half of medical school matriculants but only one-third of emergency medicine (EM) residents. Various factors may contribute to why fewer females choose the field of EM, such as the existing presence of females in the specialty.

**Objective:**

This study is a follow-up to previous work, and a survey is used to assess current residents’ attitudes and perceptions on various factors, including those relating to sex and gender on creating rank lists as medical students and in perceived effects on residency education.

**Methods:**

A web-based survey consisting of Likert scale questions regarding a variety of factors influencing a student’s decision to create a rank list and in perceived effects on residency education was sent to current EM residents in 2020.

**Results:**

Residents from 17 programs participated in the survey with an 18.2% (138/758) response rate. The most important factors in creating a rank list were the personality of residents in the program, location, and facility type. For factors specifically related to gender, respondents who answered affirmatively to whether the gender composition of residents affected the selection of a program in making a rank list were more likely to also answer affirmatively to subsequent questions related to the gender of program leadership (*P*<.001) and gender composition of attending physicians (*P*<.001). The personality of residents was also the most important factor perceived to affect residency education. For factors influencing rank list and residency education, female respondents placed higher importance on subcategories related to gender (ie, gender composition of the residents, of the program leadership, and of the attending physicians) to a significant degree compared with their male counterparts.

**Conclusions:**

Although factors such as location and resident personality show the most importance in influencing residency selection, when stratifying based on respondent sex, females tend to indicate that factors relating to gender have more influence on rank list and residency education compared with males.

## Introduction

### Background

Although females now make up more than half of medical school graduates, they compose only approximately one-third of emergency medicine (EM) residents [1,2]. It is unclear why fewer females choose to pursue EM than males. A possibility is the lack of availability of female mentorship among EM faculty. Although one could assume that female students may be more likely to attend an EM program with a higher proportion of female faculty, a study found that there was no correlation between the presence of women in leadership roles and the percentage of female residents in a program [1]. Still, women are the minority in academic medicine, with only 9.3% of the chair and 25.9% of the program director (PD) positions being held by women [1]. The presence of other female core faculty may have more influence on an applicant’s decision to choose a program. Furthermore, using resident gender distribution may not be the best surrogate for determining whether faculty gender plays a role in an applicant’s ranking decision because applicants consider many factors in their decisions and may not match with their top-choice program [2].

### Objectives

The aim of this study is to determine whether residents feel that gender distribution is an important factor when choosing a residency program. This study is a follow-up to previous work examining sex ratios across EM programs of entering years from 2014 to 2017 (Gibney et al, unpublished data, 2021). The authors approached a cross-section of the programs identified in the previous study with varied sex diversity and asked their residents to complete a survey on factors that were important in residency selection and residency education to determine what sex or gender factors were perceived as important and if any factors showed differences in importance between males and females.

Throughout this paper we use the terms *male* and *female* to discuss topics relating to sex because of the limitations of the previous study upon which this work is based, which relied on publicly available data to calculate male-female ratios. Gender, on the other hand, refers to the social construct of masculinity or femininity, or *man*, *woman*, and *nonbinary*. In the survey we created and discuss in this paper, respondents were given the opportunity to self-identify their gender as nonbinary.

## Methods

### Recruitment and Survey

The University of California, Irvine Institutional Review Board registered this study and survey as exempt, given that it was an anonymous survey with minimal risk. We designed a survey using SurveyMonkey software (Momentive). The questionnaire was not externally validated; however, it was created by several educators in EM and trialed by 5 colleagues to ensure clarity and understanding. The survey questions which were sent to participants can be found in Multimedia Appendix 1. The survey was sent to programs willing to participate further in the study, with a target population of current EM residents at Accreditation Council for Graduate Medical Education–accredited US residency programs in October-November 2019 to serve as a representative pool of residents across the country. Voluntary response sampling was used in that the survey and study information sheet were sent to the program coordinator or PD who then forwarded the survey to their residents. A chance to win a US $100 Amazon gift card was offered as an incentive, and the survey was anonymous with an option to supply an email address at the end to enter the draw.

The survey was distributed electronically through an emailed link. The link was not publicly available or advertised. Contact with participants as a group, not individuals, was through the program coordinator or PD through email. Survey data were captured automatically when participants submitted their answers. The survey was voluntary, and participants could choose to stop answering at any time. The time frame for the survey was 30 days. Items were not randomized. Adaptive questioning was used to display the Likert scale only if a participant responded *Yes* to a question about whether a factor was important to them. The survey consisted of 2 pages. The first page contained 47 demographic data and yes or no questions that displayed Likert scales for questions that were answered *yes*. The second page had 13 questions with Likert responses if answered *yes*. A completeness check was performed by making the questions mandatory. Respondents were not able to change their responses with a Back or Review step. View, participation, and completion rates were not tracked. Cookies were used by the SurveyMonkey site to assign unique respondent IDs, and there were no duplicate entries. The IP address was not used to identify duplicates. Participants did not need to register or create a survey log-in. Incomplete questionnaires could be submitted. Atypical timestamp was not used to exclude data. Items were not weighted, nor were propensity scores used. The survey methods comply with CHERRIES (Checklist for Reporting Results of Internet E-Surveys) [3]. The programs that participated in the survey included a broad spectrum of sex distributions ranging from highly male-dominated to highly female-dominated ratios (one >3:1, three 2-3:1, ten 1-2:1, one 1:1, two <1:1, male:female).

### Statistical Analysis

Categorical variables are presented as relative frequencies and continuous variables as mean (SD). We compared the association of categorical variables using a chi-square test. We compared the distribution of continuous variables among study groups by using the Mann–Whitney U test because they were not normalized. *P*<.05 was considered statistically significant. We used SPSS software (version 26.0; IBM Corp) for data analysis.

## Results

### Survey Demographics

Of the 171 EM residency programs included in our study, 17 (9.9%) agreed to participate in the follow-up survey on residency selection and education. Surveys were sent to the 758 residents, and 138 (18.2% response rate) responded. These respondents represented 17 programs across 10 states (California, Delaware, Florida, Iowa, Louisiana, Michigan, New Jersey, New York, North Carolina, and Texas) and the District of Columbia. The median age of the respondents was 28 (IQR 4) years. Of the 138 respondents, 56 (40.6%) were male, 81 (58.7%) were female, and 1 (0.7%) was nonbinary. The authors recognize the use of *male or female* terminology in the survey question self-identifying gender as a limitation of the study, and this is further addressed in the *Discussion* section.

### Decision Factors in Determining Rank Lists

**Figure 1:**
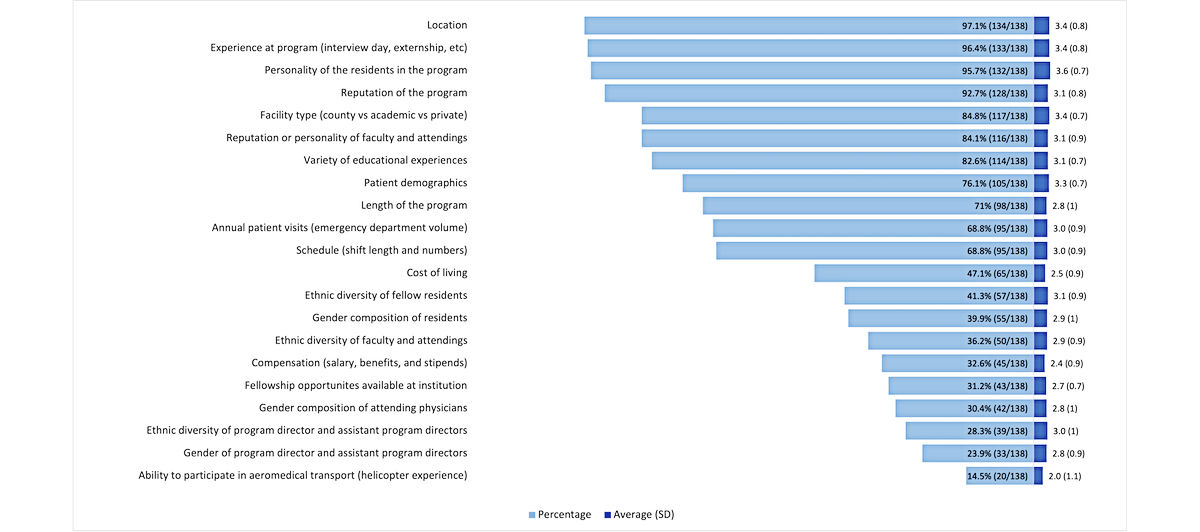
Decision factors in determining rank lists and average importance of rank list factors.

All residency selection factors and the rate at which respondents marked them as important are shown in Figure 1. The respondents noted the following as the most important factors when making their rank lists as fourth-year medical students: location (134/138, 97.1%), experience at the program (eg, interview day and externship; 133/138, 96.4%), personality of the residents in the program (133/138, 96.4%), reputation of the program (128/138, 92.7%), facility type (county vs academic vs private; 117/138, 84.8%), reputation or personality of faculty and attendings (116/138, 84.1%), variety of educational experiences (115/138, 83.3%), patient demographics (105/138, 76.1%), length of the program (98/138, 71%), annual patient visits (95/138, 68.8%), and schedule (shift length and numbers; 95/138, 68.8%).

After selecting the factors they used in determining their rank list, the respondents were asked to score on a Likert scale how important each of these factors was, with 1 being not very important and 4 being very important. Averages of the scored scales are reported in Figure 1. The single most important factor was the personality of residents in the program with a Likert scale average of 3.61 (SD 0.7). Other important factors included location (average 3.45, SD 0.8), facility type (average 3.41, SD 0.7), experience at program (average 3.36, SD 0.8), and patient demographics (average 3.34, SD 0.7).

### Decision Factors Relating to Gender and Sex Composition in Determining Rank Lists

Regarding the factors relating to gender makeup in the residency program and how they affected the rank list, the distribution of positive answers was as follows: gender composition of residents: 39.9% (55/138), 95% CI 31.6%-48.5%; gender composition of attending physicians: 30.4% (42/138), 95% CI 22.9%-38.8%; and gender of PD and assistant PDs (APDs): 23.9% (33/138), 95% CI 17.1%-31.1% (Figure 1). Gender composition of residents was more important than gender composition of PDs (*P*=.004), but the difference between the other categories listed was not statistically significant.

The survey evaluated whether the sex composition of residents, faculty, and leadership would affect residency selection if it was male or female predominant. Regarding the factors that would affect their selection of residency, of the 138 respondents, 64 (46.4%) indicated a program with only, or predominantly, male residents; 57 (41.3%) indicated a program with only, or predominantly, male faculty; and 56 (40.6%) indicated a program with only male leadership (PD or APDs). Of these respondents, 83% (53/64), 84% (48/57), and 89% (50/56), respectively, indicated at least a moderate effect on their selection of that program (Table 1).

In a subgroup analysis, respondents who answered affirmatively to whether the sex composition of residents affected selection of a program in making a rank list were more likely to also answer affirmatively to subsequent questions related to the sex of the PD and APDs (*P*<.001) and the sex composition of attending physicians (*P*<.001).

**Table 1:** Effect of male predominance among residents, faculty, and program leadership on rank list decisions.

		Values, n (%)
**Would a program that has only or predominantly male residents affect your selection of residency? How much would it affect your selection of residency? (n=60)**		
	Major effect	29 (48)
	Moderate effect	24 (40)
	Mild effect	7 (12)
**Would a program that has only or predominantly male faculty affect your selection of residency? How much would it affect your selection of residency? (n=56)**		
	Major effect	19 (34)
	Moderate effect	29 (52)
	Mild effect	8 (14)
**Would a program that has only or predominantly male residency leadership (program director and assistant or associate program director) affect your selection of residency? How much would it affect your selection of residency? (n=54)**		
	Major effect	24 (44)
	Moderate effect	26 (48)
	Mild effect	4 (7)

### Perceived Factors That Affect Residency Education

**Figure 2:**
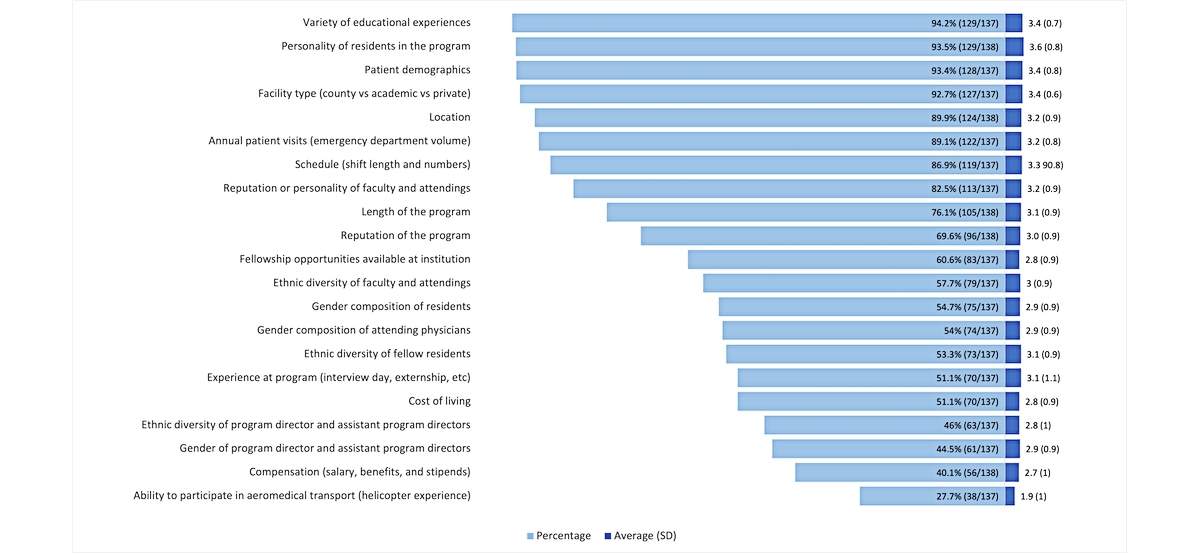
Factors perceived to affect residency education and their average importance.

Next, we examined the factors that were perceived to affect residency education. Less than half of the respondents said that compensation was a factor that affected their residency education. The three factors that were indicated to have the least influence on residency education were gender of the PD and APDs, compensation, and ability to participate in aeromedical transport. The total number of respondents in this question set varies because of an incomplete data set used in analysis. The three most important factors were variety of education experiences (129/137, 94.2%), personality of residents in the program (129/138, 93.5%), and patient demographics (128/137, 93.4%; Figure 2). Respondents were again asked to score on a Likert scale how important their selected factors were, with 1 being not very important and 4 being very important. Scored averages are shown in Figure 2. Personality of residents in the program showed the highest average score when ranked on a 4-point Likert scale (average 3.58, SD 0.8; Figure 2). Other important factors included variety of educational experiences (average 3.41, SD 0.7), facility type (average 3.4, SD 0.6), and patient demographics (average 3.4, SD 0.8; Figure 2).

### Perceived Factors Relating to Gender and Sex That Affect Residency Education

The results of factors perceived as affecting residency education followed an identical pattern to those affecting residency selection. Gender composition of residents was more important than that of attendings, which in turn was more important than gender of the PD and APDs in having a perceived effect on one’s residency education.

In the final set of questions, respondents were asked to select from >50%, >60%, >70%, >80%, and >90% to describe at what percentage they considered the faculty to be male or female predominant. The median at which a faculty was male predominant was >70%, whereas female predominant was indicated at only >60%. With respect to male- or female-predominant resident groups, the median was >60% for both. In establishing the point at which respondents felt that a faculty or residency was either male or female predominant, respondents showed a broader IQR in response to what percentage they considered to be female dominant. Respondents seemed to have a lower threshold for considering faculty or residency to be female predominant, with the IQR spanning from the minimum of >50% to >70%, whereas IQRs regarding male predominance were narrower, extending from >60% to >70%.

### Factors Related to Residency Selection and Education Stratified by Respondent Sex

There were differences in responses to certain categories based on respondent sex. A chi-square analysis was performed with *α* of .05. Factors affecting residency selection and rank lists stratified by sex are presented in Table 2. Statistically significant differences were found in females placing more importance than males on the following factors: experience at the program on interview day (*χ*^2^_1_=7.5; *P*=.006; n=137), patient demographics (*χ*^2^_1_=5.0; *P*=.03; n=137), gender composition of residents (*χ*^2^_1_=13.8; *P*<.001; n=137), gender of PD and APDs (*χ*^2^_1_=9.3; *P*=.002; n=137), gender of attending physicians (*χ*^2^_1_=13.7; *P*<.001; n=137), and ethnic diversity of fellow residents (*χ*^2^_1_=4.3; *P*=.04; n=137; Table 2).

With respect to the perceived factors that affect residency education, females placed more importance than males on the following factors: gender of PD and APDs (*χ*^2^_1_=11.6; *P*=.001; n=136), gender composition of attending physicians (*χ*^2^_1_=6.1; *P*=.01; n=136), and ethnic diversity of PD and APDs (*χ*^2^_1_=6.9; *P*=.008; n=136; Table 3). In both categories of factors affecting rank lists and factors affecting residency education, females placed higher importance on subcategories specifically related to gender (ie, gender composition of residents, gender of PD and APDs, and gender composition of attending physicians).

**Table 2:** Pearson chi-square tests comparing distribution of responses between male and female respondents for factors relating to selection of residency programs. The chi-square statistic is significant at *P*=.05 level (N=137).

Factors relating to selection of residency programs and response		Respondent sex, n (%)				Chi-square (*df*)		Significance (*P* value)
		Female (n=81)	Male (n=56)	Total (N=137)				
**Location**								
	No	3 (3.7)	1 (1.8)	4 (2.9)	0.4 (1)		—^a^	
	Yes	78 (96.3)	55 (98.2)	133 (97.1)	—		.51^b^	
**Reputation of the program**								
	No	5 (6.2)	5 (8.9)	10 (7.3)	0.4 (1)		—	
	Yes	76 (93.8)	51 (91.1)	127 (92.7)	—		.54^b^	
**Length of the program**								
	No.	27 (33.3)	12 (21.4)	39 (28.5)	2.3 (1)		—	
	Yes	54 (66.7)	44 (78.6)	98 (71.5)	—		.13	
**Compensation (salary, benefits, and stipends)**								
	No	56 (69.1)	36 (64.3)	92 (67.2)	0.4 (1)		—	
	Yes	25 (30.9)	20 (35.7)	45 (32.8)	—		.55	
**Personality of the residents in the program**								
	No	2 (2.5)	4 (7.1)	6 (4.4)	1.7 (1)		—	
	Yes	79 (97.5)	52 (92.9)	131 (95.6)	—		.19^b^	
**Experience at program (interview day, externship, etc)**								
	No	0 (0)	5 (8.9)	5 (3.6)	7.5 (1)		—	
	Yes	81 (100)	51 (91.1)	132 (96.4)	—		.006^b^	
**Fellowship opportunities available at institution**								
	No	55 (67.9)	39 (69.6)	94 (68.6)	0.5 (1)		—	
	Yes	26 (32.1)	17 (30.4)	43 (31.4)	—		.83	
**Patient demographics**								
	No	14 (17.3)	19 (33.9)	33 (24.1)	5.0 (1)		—	
	Yes	67 (82.7)	37 (66.1)	104 (75.9)	—		.03	
**Variety of educational experiences**								
	No	11 (13.6)	13 (23.2)	24 (17.5)	2.1 (1)		—	
	Yes	70 (86.4)	43 (76.8)	113 (82.5)	—		.15	
**Gender composition of residents**								
	No	38 (46.9)	44 (78.6)	82 (59.9)	13.8 (1)		—	
	Yes	43 (53.1)	12 (21.4)	55 (40.1)	—		<.001	
**Gender of program director and assistant program directors**								
	No	54 (66.7)	50 (89.3)	104 (75.9)	9.3 (1)		—	
	Yes	27 (33.3)	6 (10.7)	33 (24.1)	—		.002	
**Schedule (shift length and numbers)**								
	No	23 (28.4)	20 (35.7)	43 (31.4)	0.8 (1)		—	
	Yes	58 (71.6)	36 (64.3)	94 (68.6)	—		.36	
**Gender composition of attending physicians**								
	No	47 (58)	49 (87.5)	96 (70.1)	13.7 (1)		—	
	Yes	34 (42)	7 (12.5)	41 (29.9)	—		<.001	
**Annual patient visits (emergency department volume)**								
	No	26 (32.1)	16 (28.6)	42 (30.7)	0.2 (1)		—	
	Yes	55 (67.9)	40 (71.4)	95 (69.3)	—		.66	
**Cost of living**								
	No	46 (56.8)	26 (46.4)	72 (52.6)	1.4 (1)		—	
	Yes	35 (43.2)	30 (53.6)	65 (47.4)	—		.23	
**Facility type (county vs academic vs private)**								
	No	14 (17.3)	7 (12.5)	21 (15.3)	0.6 (1)		—	
	Yes	67 (82.7)	49 (87.5)	116 (84.7)	—		.45	
**Ethnic diversity of fellow residents**								
	No	42 (51.9)	39 (69.6)	81 (59.1)	4.3 (1)		—	
	Yes	39 (48.1)	17 (30.4)	56 (40.9)	—		.04	
**Ethnic diversity of faculty and attendings**								
	No	48 (59.3)	40 (71.4)	88 (64.2)	2.1 (1)		—	
	Yes	33 (40.7)	16 (28.6)	49 (35.8)	—		.14	
**Ethnic diversity of program director and assistant program directors**								
	No	55 (67.9)	44 (78.6)	99 (72.3)	1.9 (1)		—	
	Yes	26 (32.1)	12 (21.4)	38 (27.7)	—		.17	
**Ability to participate in aeromedical transport (helicopter experience)**								
	No	71 (87.7)	46 (82.1)	117 (85.4)	0.8 (1)		—	
	Yes	10 (12.3)	10 (17.9)	20 (14.6)	—		.37	
**Reputation or personality of faculty and attendings**								
	No	10 (12.3)	11 (19.6)	21 (15.3)	1.4 (1)		—	
	Yes	71 (87.7)	45 (80.4)	116 (84.7)	—		.24	

^a^Not available.

^b^More than 20% of the cells in this subtable have expected cell counts <5. Chi-square results may be invalid.

**Table 3:** Pearson chi-square tests comparing distribution of responses between male and female respondents for factors relating to education in residency programs. The chi-square statistic is significant at *P*=.05 level (N=136-137).

Factors relating to education in residency programs and response		Respondent sex, n (%)				Chi-square (*df*)		Significance (*P* value)
		Female (n=81)	Male (n=56)	Total (N=136-137)				
**Location**								
	No	9 (11.1)	5 (8.9)	14 (10.2)	0.2 (1)		—^a^	
	Yes	72 (88.9)	51 (91.1)	123 (89.8)	—		.68	
**Reputation of the program**								
	No	20 (24.7)	22 (39.3)	42 (30.7)	3.3 (1)		—	
	Yes	61 (75.3)	34 (60.7)	95 (69.3)	—		.07	
**Length of the program**								
	No	23 (28.4)	10 (17.9)	33 (24.1)	2.0 (1)		—	
	Yes	58 (71.6)	46 (82.1)	104 (75.9)	—		.16	
**Compensation (salary, benefits, and stipends)**								
	No	48 (59.3)	34 (60.7)	82 (59.9)	0.3 (1)		—	
	Yes	33 (40.7)	22 (39.3)	55 (40.1)	—		.86	
**Personality of the residents in the program**								
	No	4 (4.9)	5 (8.9)	9 (6.6)	0.9 (1)		—	
	Yes	77 (95.1)	51 (91.1)	128 (93.4)	—		.35^b^	
**Experience at program (interview day, externship, etc)^c^**								
	No	36 (45)	30 (53.6)	66 (48.5)	1.0 (1)		—	
	Yes	44 (55)	26 (46.4)	70 (51.5)	—		.33	
**Fellowship opportunities available at institution^c^**								
	No	31 (38.8)	22 (39.3)	53 (39)	0.0 (1)		—	
	Yes	49 (61.3)	34 (60.7)	83 (61)	—		.95	
**Patient demographics^c^**								
	No	5 (6.3)	4 (7.1)	9 (6.6)	0.0 (1)		—	
	Yes	75 (93.8)	52 (92.9)	127 (93.4)	—		.84^b^	
**Variety of educational experiences^c^**								
	No	3 (3.8)	4 (7.1)	7 (5.1)	0.8 (1)		—	
	Yes	77 (96.3)	52 (92.9)	129 (94.9)	—		.38^b^	
**Gender composition of residents^c^**								
	No	31 (38.8)	31 (55.4)	62 (45.6)	3.7 (1)		—	
	Yes	49 (61.3)	25 (44.6)	74 (54.4)	—		.06	
**Gender of program director and assistant program directors^c^**								
	No	35 (43.8)	41 (73.2)	76 (55.9)	11.6 (1)		—	
	Yes	45 (56.3)	15 (26.8)	60 (44.1)	—		.001	
**Schedule (shift length and numbers)^c^**								
	No	8 (10)	10 (17.9)	18 (13.2)	1.8 (1)		—	
	Yes	72 (90)	46 (82.1)	118 (86.8)	—		.18	
**Gender composition of attending physicians^c^**								
	No	30 (37.5)	33 (58.9)	63 (46.3)	6.1 (1)		—	
	Yes	50 (62.5)	23 (41.1)	73 (53.7)	—		.01	
**Annual patient visits (emergency department volume)^c^**								
	No	8 (10)	7 (12.5)	15 (11)	0.2 (1)		—	
	Yes	72 (90)	49 (87.5)	121 (89)	—		.65	
**Cost of living^c^**								
	No	42 (52.5)	24 (42.9)	66 (48.5)	1.2 (1)		—	
	Yes	38 (47.5)	32 (57.1)	70 (51.5)	—		.27	
**Facility type (county vs academic vs private)^c^**								
	No	8 (10)	2 (3.6)	10 (7.4)	2.0 (1)		—	
	Yes	72 (90)	54 (96.4)	126 (92.6)	—		.16^b^	
**Ethnic diversity of fellow residents^c^**								
	No	32 (40)	31 (55.4)	63 (46.3)	3.1 (1)		—	
	Yes	48 (60)	25 (44.6)	73 (53.7)	—		.08	
**Ethnic diversity of faculty and attendings^c^**								
	No	30 (37.5)	28 (50)	58 (42.6)	2.1 (1)		—	
	Yes	50 (62.5)	28 (50)	78 (57.4)	—		.15	
**Ethnic diversity of program director and assistant program directors^c^**								
	No	36 (45)	38 (67.9)	74 (54.4)	6.9 (1)		—	
	Yes	44 (55)	18 (32.1)	62 (45.6)	—		.008	
**Ability to participate in aeromedical transport (helicopter experience)^c^**								
	No	58 (72.5)	40 (71.4)	98 (72.1)	0.2 (1)		—	
	Yes	22 (27.5)	16 (28.6)	38 (27.9)	—		.90	
**Reputation or personality of faculty and attendings^c^**								
	No	13 (16.3)	11 (19.6)	24 (17.6)	0.3 (1)		—	
	Yes	67 (83.8)	45 (80.4)	112 (82.4)	—		.61	

^a^Not available.

^b^More than 20% of the cells in this subtable have expected cell counts <5. Chi-square results may be invalid.

^c^Sample size changes from 137 to 136 because of incomplete data set used in analysis.

## Discussion

### Principal Findings

We hypothesized that program leadership may influence a student’s rank list in a way that tends to favor the propagation of a similar sex distribution among residents. Although medical school matriculation rates are nearly equal between male and female students, the percentage of females represented in academic medicine remains disparate [4]. Within EM, the Association of American Medical Colleges reports that as of 2015, women made up only 33% of the EM faculty, with only 17% representation seen among full professors [4]. Potential reasons that account for the disparity include a lack of mentors, greater work–life balance prioritization, and gender discrimination and bias [5]. Our findings on gender diversity within EM residency leadership was consistent with previous data showing 76% of the programs with male directors [6]; however, direct influence of the PD and faculty gender had not previously been evaluated.

To avoid bias regarding the topic of the survey, we included numerous aspects unrelated to gender in the questionnaire to allow survey respondents to appropriately consider all relevant aspects of selection and education. Many of the topics were similar to those found in the National Resident Matching Program (NRMP) Applicant Survey, and the factors that were considered most important were similar to those found in the 2019 NRMP Applicant Survey results for EM [7].

As a cohort, our survey results suggest that faculty and residency leadership gender makeup have minimal effect on residency selection. However, when stratified by respondent sex, our study found that female respondents viewed gender distribution of PD and APDs and attendings as influencing their residency selection and education more than their male counterparts. In programs with lower male–female ratios in leadership, incoming female residents may rank these institutions higher for the potential for female mentorship. A previous study on sex distribution in radiology residencies showed that programs with a female PD had a higher concentration of female residents [8], and a similar study in EM showed no difference [1]. However, in our study, these factors do not seem as important as others.

The relatively short time a medical student spends at a given program during a rotation or interview necessitates that the student must make inferences about how their own experience will be if matched into that program. The concept of homophily—the tendency to favor those like oneself—can potentially explain this trend. In the 8 programs with a higher percentage of female residents than male residents, program leadership also reflected a low male-female ratio, and all these programs had at least one female represented in leadership. In addition, residents who seek to perform research and achieve publication throughout their training may also place high value on such opportunities when determining rank lists. Although homophily has been described as being more commonly observed among females, the phenomenon has been observed in the publication realm and shown to be stronger in male journal editors [9]. Within EM publications, only 26% of the first authorships are female [10]. A study in 2011 reported that only 15.9% of the editors-in-chief and 17.5% of the editorial board members of 60 top-ranking journals were female [11]. Thus, if seeking research opportunities during residency, incoming female trainees may gravitate toward programs that visibly promote advancement and career development that is more favorable toward females than toward programs with predominantly or exclusively male leadership.

Sex distributions among current residents seem to play a role in creating a rank list. In residency programs that are predominantly male, incoming female residents may perceive a lack of fit in these programs because they do not see as many female colleagues. Our study shows that the primary gender makeup affecting applicants’ ranking of programs was that of the residents. Female respondents indicated that the gender composition of residents influenced their rank list and education more than their male counterparts. Incoming female residents may view programs with a higher female presence more favorably because they can see themselves being successful. The same can be said for male residents as well. However, because EM as a specialty is predominantly male, females may be influenced to rank programs with a greater percentage of female residents higher because they see other females being successful as a resident at that institution. As Bandura [12] describes in his concept of self-efficacy, observing people similar to oneself be successful increases one’s belief in achieving the same success. Therefore, in the recruitment of residents, programs should examine their own sex and gender makeup to determine ways to address cognitive biases that may result from a skewed distribution.

### Limitations

Limitations to our study include having a low survey response rate (138/758, 18.2%) and low overall sample size; however, our sample was well distributed in terms of geographic location and age of respondents. Furthermore, for the non–gender-related items, the results are similar to the 2019 NRMP Applicant Survey results, suggesting that there may not be significant bias from the response rate. In addition, there is a factor of retrospective recall in that the residents were surveyed after matriculation, rather than at the time of ranking decisions. There is also response bias with having more females than males take the survey; therefore, the factors identified in our study may represent elements more important to females than to males. There is also the uncontrollable wildcard inherent in the NRMP. Programs may rank incoming residents with a nearly even male-female split, but, depending on algorithms and student choice, the sex ratios expected may not match the outcome. However, it is still important to recognize the possibility for existing sex distributions among residents and program leadership to influence a fourth-year medical student’s decision to rank that program.

Another limitation of our study is the use of *male* and *female* as response options in self-identifying gender in our survey. Future work would benefit from clear distinctions in demographic data with separate questions for respondent sex (eg, male or female) and gender (eg, man, woman, and nonbinary) to capture characteristics of respondents more accurately.

### Comparison With Prior Work

More recently, Mannix et al [13] examined sex distribution among chief residents in EM. The group found that females have increased representation among chief residents compared with their overall proportion among EM residents, with females and males having a similar presence in the chief positions. Our study did not examine the perceptions of having female representation among chief residents, although Mannix et al [13] also suggest that increased numbers of female chief residents will help bridge the sex gap in academic medicine and program leadership observed currently. Our data similarly show that current female residents place higher value on female leadership among PDs and APDs than male residents.

DeSantis and Marco [14] previously reported that friendliness, environment, and interview day experience were the top 3 factors that were important to residents in selecting their program. Our study echoed similar results, with aspects such as location, experience at the program, and personality of the residents having a significant influence on a resident’s choice and education. As Laskey and Cydulka [15] reported in a 2009 study, female residents valued opportunities to serve specific populations as more important than their male counterparts, and in our study, we also found a similar trend with regard to patient demographics as being an influencing factor in rank list and residency education.

### Conclusions

Follow-up surveys to quantify the importance of sex and gender in residency selection showed that other factors such as location, interview day experience, personality of residents, and educational experiences were rated as much more important than gender differences within a program. In stratifying results based on male and female respondents, female respondents tended to indicate that factors relating to gender had more influence on their decisions in creating a rank list and perceptions of residency education more often than male respondents.

## Appendix

Multimedia Appendix 1 Survey questions sent to participants.

## Abbreviations

APD: assistant program director
CHERRIES: Checklist for Reporting Results of Internet E-Surveys
EM: emergency medicine
NRMP: National Resident Matching Program
PD: program director
