# Supplementary material for: Influence of Factors Relating to Sex and Gender on Rank List Decisions and Perceptions of Residency Training: Survey Study
Source: JMIR Med Educ. 2022 Apr 5;8(2):e33592. doi: 10.2196/33592 (PMC9019614; doi:10.2196/33592)
Supplement: Multimedia Appendix 1 [file mededu_v8i2e33592_app1.docx]

**Multimedia Appendix 1.** Survey questions sent to participants.

Name of your residency:

What Residency Year are you currently in?

PGY 1-4

What is your Gender

M/F/non-binary

What is your age

Open answer

What is your race? (NIH definition)

White

Asian

Black or African American

Hispanic or Latino

American Indian or Alaska Native

Did the below factors affected selection of your residency program WHEN you were a fourth year medical student making your rank list:

Yes or no, then Likert scale if yes

1. Location
2. Reputation of the program
3. Length of program
4. Compensation (salary, benefits, stipend)
5. Personality of residents in the program
6. Program director
7. Experience at program (interview day, externship)
8. Fellowship opportunities available at institution
9. Patient demographics
10. Variety of educational experiences (other sites, rotations)
11. Gender composition of residents
12. Gender of program director and assistant program directors
13. Schedule (shift lengths and numbers)
14. Gender composition of attending physicians
15. Annual patient visits (ED volume)
16. Cost of living
17. Facility type (county vs. academic vs. private)
18. Ethnic diversity of fellow residents
19. Ethnic diversity of faculty/attendings
20. Ethnic diversity of program director and assistant program directors
21. Ability to participate in aeromedical transport (helicopter experience)
22. Reputation or personality of faculty/attendings

Do you feel the below factors affect your residency education.

Yes or no, then Likert scale if yes

1. Location
2. Reputation of the program
3. Length of program
4. Compensation (salary, benefits, stipend)
5. Personality of residents in the program
6. Program director
7. Experience at program (interview day, externship)
8. Fellowship opportunities available at institution
9. Patient demographics
10. Variety of educational experiences (other sites, rotations)
11. Gender composition of residents
12. Gender of program director and assistant program directors
13. Schedule (shift lengths and numbers)
14. Gender composition of attending physicians
15. Annual patient visits (ED volume)
16. Cost of living
17. Facility type (county vs. academic vs. private)
18. Ethnic diversity of fellow residents
19. Ethnic diversity of faculty/attendings
20. Ethnic diversity of program director and assistant program directors
21. Ability to participate in aeromedical transport (helicopter experience)
22. Reputation or personality of faculty/attendings

END PAGE 1, cannot go back to change answers

BEGIN PAGE 2:

Does the gender make-up of your residency leadership affect your education?

Yes No

How much does it affect your education: Likert scale 1-5

Does the gender make-up of your faculty affect your education?

Yes No

How much does it affect your education: Likert scale 1-5

Does the gender make-up of your fellow residents affect your education?

Yes No

How much does it affect your education: Likert scale 1-5

Would a program that has only male residency leadership (program director and APD) affect your desire to attend that residency?

Yes No

How much would it affect your desire to attend: Likert 1-5

Would a program that has only female residency leadership (program director and APD) affect your desire to attend that residency?

Yes No

How much would it affect your desire to attend: Likert 1-5

Would a program that has only or predominantly male faculty affect your desire to attend that residency?

Yes No

How much would it affect your desire to attend: Likert 1-5

At what percent do you consider a faculty predominantly male?

>50% >60% >75% >80% >90%

Would a program that has only or predominantly female faculty affect your desire to attend that residency?

Yes No

How much would it affect your desire to attend: Likert 1-5

At what percent do you consider a faculty predominantly female?

>50% >60% >75% >80% >90%

Would a program that has only or predominantly male residents affect your desire to attend that residency?

Yes No

How much would it affect your desire to attend: Likert 1-5

At what percent do you consider a residency predominantly male?

>50% >60% >75% >80% >90%

Would a program that has only or predominantly female residents affect your desire to attend that residency?

Yes No

How much would it affect your desire to attend: Likert 1-5

At what percent do you consider a residency predominantly male?

>50% >60% >75% >80% >90%

END PAGE 2, submit answers

PAGE 3: Email (separated from main survey) for optional collection of email addresses for entry to win Amazon gift card incentive.
